# Biotechnological Potential of Quorum Quenching Bacterial Strains Isolated from *Perca fluviatilis*

**DOI:** 10.3390/ani16091339

**Published:** 2026-04-27

**Authors:** Dmitry Andreevich Dokolin, Ilya Vasilevich Zlobin, Maksim Nikolaevich Sokolov, Andrey Sergeevich Sysuev, Aleksandra Aleksandrovna Payuta, Ekaterina Aleksandrovna Flerova, Yuliya Vladimirovna Zaitseva

**Affiliations:** Laboratory of Molecular Genetics and Biotechnology, Yaroslavl State University, Yaroslavl 150003, Yaroslavl Region, Russia; melsudbi@yandex.ru (M.N.S.); andrey-sysuek@yandex.ru (A.S.S.); a.payuta@mail.ru (A.A.P.); katarinum@mail.ru (E.A.F.); zjv9@mail.ru (Y.V.Z.)

**Keywords:** *Rhodococcus*, *Aeromonas*, probiotics, Quorum Sensing, AHL, Quorum Quenching, lactonases, genome potential

## Abstract

Quorum Quenching (QQ) is a mechanism that disrupts Quorum Sensing (QS) signaling systems, which regulate gene expression based on bacterial population density. Many fish pathogens, such as *Aeromonas*, utilize QS systems to regulate the expression of their virulence factors. Disrupting these systems using QQ is a promising approach for infection control in aquaculture and may provide a safe alternative to antibiotics. Therefore, identifying microorganisms with QQ activity is a relevant task in agricultural microbiology and veterinary medicine. This study examines the identification of isolates with QQ activity in the microbial community of perch and assesses their probiotic potential for the prevention of aeromonosis. In this study, we isolated 32 strains of microorganisms capable of degrading N-acylhomoserine lactone (AHL), six of which exhibited stable QQ activity. Five strains were found to belong to the genus *Rhodococcus*, and one strain to the genus *Exiguobacterium*. The selected strains were tested for the enzymatic/non-enzymatic and intra-/extracellular QQ activity, pathogen growth inhibition, biofilm formation, and hemolytic activity, as well as growth ability under various environmental conditions (salinity, pH, bile acids, and temperature). Based on the results of these tests, the *R. erythropolis* PFS1.20 strain was selected as the most promising probiotic. The genomic analysis revealed that the studied strain contains genes encoding QQ enzymes, siderophore biosynthesis clusters, osmoprotectors, and compounds with antimicrobial properties. These results indicate the high probiotic potential of the *R. erythropolis* PFS1.20.

## 1. Introduction

Many bacteria are capable of intercellular communication within populations through Quorum Sensing (QS) systems, which function by the release of signaling molecules into the environment. QS enables bacteria to regulate gene expression in response to changes in population density. Gram-negative bacteria use N-acylhomoserine lactones (AHLs) as QS signals. Generally, AHL molecules consist of a lactone ring linked to an acyl side chain via an amide bond [1]. Variations in the length of the acyl chain and the presence of substituent groups within the AHL molecule ensure signal specificity across different phylogenetic groups [2].

QS systems play a significant role in regulating pathogenicity. For instance, in the genus *Aeromonas*, QS systems regulate the synthesis of exopolysaccharides, motility, and plasmid transfer during conjugation [3]. These microorganisms cause a disease in fish collectively referred to as “aeromonosis”, characterized by symptoms such as gill hemorrhages, exophthalmia, fin rot, abdominal distension, and ulcers on the body surface [3].

Disrupting the functioning of these systems could serve as a strategy for controlling the spread of bacterial infections. Processes that interfere with QS are termed Quorum Quenching (QQ) [4,5,6]. Unlike antibiotics, QQ does not kill bacterial cells and exerts less selective pressure on pathogens [7]. Enzymatic degradation of AHL molecules is considered one of the most promising QQ approaches.

**Figure 1:**
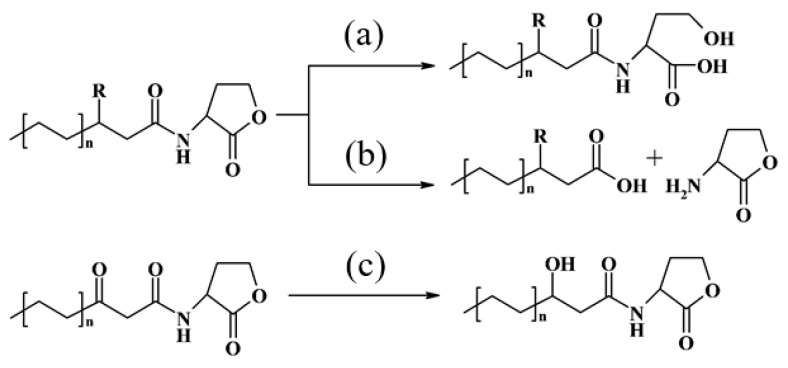
Pathways of AHL enzymatic destruction: (**a**) AHL destruction by lactonases; (**b**) AHL destruction by acylases; (**c**) AHL modification by oxidoreductases.

To date, three groups of enzymes responsible for AHL inactivation have been identified: lactonases, acylases (amidases), and oxidoreductases [8]. Lactonases catalyze the hydrolysis of ester bonds within the lactone ring (Figure 1a). Acylases catalyze the hydrolysis of amide bonds, yielding homoserine lactone and a fatty acid (Figure 1b). Oxidoreductases catalyze redox reactions (Figure 1c).

Phylogenetically diverse bacteria are capable of disrupting AHL-based Quorum Sensing. Isolates belonging to the phyla Pseudomonadota (genera *Acinetobacter*, *Achromobacter*, *Agrobacterium*, *Brevundimonas*, *Comamonas*, *Klebsiella*, *Ochrobactrum*, *Pseudomonas*, *Ralstonia*, *Shewanella*, *Sphingomonas*, *Stenotrophomonas*, and others), Bacteroidota (*Chryseobacterium*, *Flaviramulus*, and *Tenacibaculum*), Cyanobacteriota (*Nostoc*), Actinomycetota (genera *Arthrobacter*, *Microbacterium*, *Nocardioides*, *Rhodococcus*, *Staphylococcus*, and *Streptomyces*), Deinococcota (*Deinococcus*), and Bacillota (*Bacillus*, *Solibacillus*) have been isolated and characterized as AHL-degrading bacteria [9]. Interest in the discovery of novel organisms exhibiting QQ activity is driven by their broad potential for biotechnological applications, including aquaculture. Successful examples of using enzymes to combat fish pathogens have been reported. For instance, the addition of purified AiiA lactonase, isolated from the strain *Bacillus* sp. QSI-1, to the feed of *Danio rerio* and *Carassius auratus,* reduced mortality caused by *A. hydrophila* infection [10]. The YtnP lactonase from *B. licheniformis* T-1 inhibited biofilm formation and effectively reduced the pathogenicity of *A. hydrophila* ATCC 7966 [11]. In similar work, it was demonstrated that YtnP lactonase presented superior enzyme structural stability and higher degradation efficiency of AHLs than AiiA lactonase under the effects of pH, temperature, and performed better on short-chain and 3-O-substituted AHSLs [12]. However, the production of purified enzymes is a relatively costly process. The strategy of utilizing probiotic microorganisms that produce QQ enzymes appears more feasible. The ability to degrade AHLs, as an important criterion for selecting probiotic isolates for aquaculture, has been widely demonstrated for bacteria of the genus *Bacillus* [11,13]. *Bacillus* isolates *B. cereus* KM1 and *B. velezensis* KM2, isolated from mangrove sediments, have displayed probiotic characteristics. Both showed good antagonistic activity against aquatic pathogens such as *Aeromonas hydrophila*, *Vibrio parahaemolyticus*, and *Streptococcus agalactiae*. In addition, both isolates showed the ability to degrade more than 70% of the initial AHL. Also, isolates were found to tolerate varying concentrations of bile salts and extreme pH conditions of fish gastrointestinal juice [14]. Despite the considerable phylogenetic diversity of bacteria possessing QQ activity, their probiotic potential remains poorly studied. Microorganisms associated with aquatic organisms are considered a particularly promising source of probiotics for aquaculture.

In a recent study [15], a total of fifty-two *Streptomyces* strains were isolated from aquaculture pond sediment and fish intestines and were identified. Half of the isolated strains exhibited varying degrees of antagonistic activity against a variety of common pathogenic bacteria in aquaculture, such as *Aeromonas hydrophila*, *Pseudomonas aeruginosa*, *Vibrio parahemolyticus*, *Staphylococcus aureus*, and *Streptococcus agalactiae*. Nine strains showed clear QS inhibition activity in tests with biosensors.

The aim of this study was to isolate novel bacterial strains exhibiting QQ activity from the skin of *P. fluviatilis* and to assess their probiotic potential for the prevention of aeromonosis in aquaculture.

## 2. Materials and Methods

### 2.1. Sample Collection and Preparation

The strains were isolated from the skin of perch (*Perca fluviatilis*). Fish were caught in the Volga River near the mouth of the Voronovka River (Russia, Yaroslavl Region; coordinates: 57.940901 N, 38.279680 E). For sampling, scales and mucus were scraped from the surface of the body along the lateral line. The material was homogenized in sterile water, plated on MPA-containing plates, and incubated at 28 °C for 72 h. For further analysis, individual colonies were selected.

### 2.2. Qualitative Analysis of QQ

The *Chromobacterium violaceum* strain CV026 biosensor [16] was used to detect QS signal molecules. Testing was carried out according to the method described in [17]. Cultures of *C. violaceum* CV026 and *Aeromonas* sp. IR145 (AHL producer) were grown separately on LB agar plates. The biosensor and AHL producer were applied to the LB agar plate in two parallel streaks, approximately 10 mm apart. The test strain was applied in a parallel streak between the biosensor and AHL producer. The plates were incubated for 48 h at 28 °C. The absence of biosensor staining indicated the presence of QQ activity in the test strains.

### 2.3. Quantitative Analysis of QQ

For quantitative assessment of QQ, an overnight culture of the selected strains was mixed with LB medium containing 10 μM N-hexanoyl-L-homoserine lactone (C6-AHL) and 50 mM propane sulfonic acid (MOPS) at a 1:9 ratio and incubated for 24 h at 28 °C. E. coli BL21 was used as a negative control. Then, cultures were centrifuged at 10,000 rpm, supernatant was sampled. The *C. violaceum* CV026 biosensor was evenly spread onto the surface of a plate containing LB agar, and wells were cut using a sterile drill. 20 μL of supernatant were added into wells and incubated at 28 °C for 24 h. The degree of AHL degradation was assessed by a decrease in the diameter of the biosensor staining zone around the well compared to the control. C6-AHL (Sigma-Aldrich, St. Louis, MO, USA) was used as a standard.

### 2.4. Assay of QQ Enzymatic Activity

Fresh bacterial cultures were heated at 95 °C for 20 min to inactivate proteins. Then, 100 mM C6-AHL was added, and samples were incubated for 24 h at 28 °C. The *C. violaceum* CV026 biosensor was pre-seeded onto LB agar plates, wells were cut with a sterile drill, and 50 µL of heat-treated suspensions were added. Plates were incubated for 24 h at 28 °C. Restoration of CV026 violacein production after heat treatment (i.e., loss of AHL-degrading activity) indicated that QQ activity was protein-dependent and therefore enzymatic.

### 2.5. Cellular/Extracellular Localization of QQ Activity

Fresh cultures of QQ isolates were centrifuged (6000× *g*, 15 min), and the supernatants were filtered through a 0.22 µm filter to obtain cell-free culture fluids. Each sample was supplemented with 100 mM C6-AHL and incubated for 24 h at 28 °C. CV026 was plated on LB agar, wells were cut in the agar with a sterile drill, and 50 µL of each culture fluid was added. After 24 h incubation at 28 °C, the absence of AHL degradation in cell-free supernatants indicated that QQ activity was cell-associated (intracellular) rather than extracellular.

### 2.6. Strain Identification

Genomic DNA was isolated using the Extract DNA kit according to the manufacturer’s instructions (Eurogen, Moscow, Russia). The 16S rRNA genes were amplified using universal prokaryotic primers: 16S-9F (5′-GAGTTTGATCCTGGCTCAG) and 16S-1512R (5′-ACGGCTACCTTGTTACGACTT). Bacteria were identified based on 16S rRNA gene primary nucleotide sequence analysis using the GenBank database and BLAST 2.12.0 software http://blast.ncbi.nlm.nih.gov/Blast.cgi (accessed on 5 May 2025). A phylogenetic tree was constructed using the Maximum Likelihood (ML) method with MEGA 12.1 software [18].

### 2.7. Search for QQ Genes in Isolates

A search for the QQ genes *qsdA* and *jydB* in the selected strains was performed using the PCR method. Search for conserved regions of the QQ genes multiple sequence alignment using the CLUSTAL Omega algorithm was performed (https://www.ebi.ac.uk/jdispatcher/msa/clustalo?stype=dna, accessed on 7 April 2025). Using GeneRunner software (version 6.5.52x64 Beta), primers for the *qsdA* and *jydB* genes were designed: qsdA-F (5′-CAGTACAAACCGTTCGTGG-3′), qsdA-R (5′-TAGATCAACCATCGGCTCG-3′), jydB-F (5′-GACAACCACCAAGATCGAAACCC-3′), jydB-R (5′-AGGTCCAGAGTGATCATGCGGTAG-3′). PCR conditions: pre-denaturation at 95 °C for 4 min; denaturation at 95 °C for 1 min, annealing at 60 °C for 1 min, elongation at 72 °C for 30 s, 30 cycles; final elongation at 72 °C for 5 min. Amplicons were detected by electrophoresis in 1.5% agarose gel.

### 2.8. Evaluation of the Ability of QQ Strains to Inhibit Aeromonas Growth In Vitro

The ability of the studied strains to inhibit growth of *Aeromonas in vitro* conditions was tested by co-cultivation as described [19]. The strains *A. salmonicida* A1, *A. salmonicida* W70, *A. piscicola* GPR23, and *A. bestiarum* VR410 from the laboratory collection were used as model strains. Selected strains with QQ activity were plated on LB agar medium and incubated for 24 h at 28 °C. Agar discs containing the test strains were then drilled out and placed into plates, containing the chosen *Aeromonas* strains. The plates were then incubated at 28 °C for 24 h. The ability of the test strains to inhibit the growth of *Aeromonas* bacteria in vitro was assessed by the presence of a growth inhibition zone around the corresponding disc.

### 2.9. Evaluation of the Ability of QQ Strains to Inhibit the Hemolytic Properties of Aeromonas

The ability of the selected strains to inhibit the hemolytic activity in *Aeromonas* bacteria was determined using a modified method described by [20]. AHL degrading strains and *Aeromonas* strains were incubated in LB medium until suspension cultures with an OD_600_ of 1.0 were obtained. A mixture of 500 μL of 5% red blood cell concentrate in 0.9% NaCl, 450 μL of the test strain, and 50 μL of the *Aeromonas* strain was then prepared. Co-cultivation was carried out for 6 h, after which the tubes were centrifuged at 4000 rpm for 2 min. The intensity of hemolysis was determined by measuring the optical density of the supernatant at a wavelength of 543 nm. A mixture of 500 μL of red blood cell concentrate and 500 μL of LB medium was used as a negative control. Red blood cell concentrate lysed in deionized water was used as a positive control.

### 2.10. Biofilm Suppression Ability of QQ Strains

To assess the ability of the studied microbial strains to suppress biofilm formation in *Aeromonas*, a modified version of protocol [21] was used. The strains were grown in LB medium until a cell suspension with an optical density of OD_600_ = 1.0 was obtained. The suspension cultures of the studied microorganisms were then lysed using ultrasound. Then, 100 µL of *Aeromonas* culture (OD_600_ = 0.1) and 100 µL of strain lysate were added to the wells of a 96-well plate. A mixture of 100 µL of *Aeromonas* culture and 100 µL of LB medium was used as a positive control in the experiment, and 200 µL of LB was used as a negative control. The plate was incubated in a shaker incubator at 150 rpm and 28 °C for 24 h. After incubation, the optical density of the well contents was measured using a BioRad iMark plate reader at 600 nm. Biofilm formation was then assessed using a standard plate assay using crystal violet dye [22].

### 2.11. Evaluation of the Ability of QQ Isolates to Grow at Different Salinities

To determine the ability of QQ isolates to grow at different salinity level, 9 mL of LB medium with 0.5%, 1%, 2%, and 3% NaCl were inoculated with 1 mL of suspension cultures with an OD_600_ = 1.0. CFUs were then plated in triplicate, and the OD_600_ was measured for each experimental variant. Measurements were performed three times: at the start of the experiment, after 24 h, and after 48 h.

The specific growth rate of microorganisms was calculated using the formula:

µ = (ln(N1) − ln(N0)) ÷ (t1 − t0)

where µ is the growth rate of the culture (h^−1^), N1 is the number of viable microorganisms (CFU/mL) at time t1, and N0 is the number of viable microorganisms (CFU/mL) at time t0.

### 2.12. Evaluation of the Ability of QQ Strains to Grow Under Environmental Factors

To evaluate the ability of the studied strains to grow at different pH levels, 1 mL of culture (OD_600_ = 1.0) was mixed with 9 mL of LB medium with pH values of 3.0, 5.0, 7.0, and 9.0. OD_600_ was measured at the beginning of the experiment, after 24 h, and after 48 h.

To assess the strains ability to grow in the presence of bile salts, 1 mL of a suspension culture of the tested strain with an OD_600_ = 1.0 was mixed with 9 mL of LB medium containing 0%, 0.25%, 0.5%, 0.75%, and 1% sodium deoxycholate. The tubes with mixture were incubated at 28 °C for 48 h. During the incubation, changes in OD_600_ were monitored for each experimental variant at 0, 24, and 48 h after inoculation.

To assess the strains growth ability at different ambient temperatures, 1 mL of overnight cultures (OD_600_ = 0.6) were inoculated into 9 mL of LB medium and incubated at different temperatures (8 °C, 15 °C, 28 °C, and 37 °C) for 48 h. The growth rate of the isolates at each temperature was assessed based on the optical density of the medium at a wavelength of 600 nm 24 h and 48 h after inoculation.

The specific growth rate of the culture was calculated using the formula [23]:

µ = (ln(D1) − ln(D0)) ÷ (t1 − t0)(1)

where µ is the specific growth rate of the culture (h^−1^), D1 is the OD_600_ at time t1, and D0 is the OD_600_ at time t0.

### 2.13. Antibacterial Resistance

The resistance of the studied strains to antimicrobial drugs (AMD) was tested using the standard disk diffusion method [24]. The following AMD disks (HiMedia, Nashik, Dindori, India) were used: ampicillin (10 μg/mL), kanamycin (30 μg/mL), rifampin (5 μg/mL), tetracycline (30 μg/mL), and gentamicin (10 μg/mL).

### 2.14. Genome Sequencing and Assembly

Genomic DNA was extracted using the Monarch Genomic DNA Purification Kit (New England Biolabs, Ipswich, MA, USA). The concentration and quality of gDNA were assessed using spectrophotometry and fluorimetry. The genomic library for sequencing was prepared using the Oxford Nanopore Ligation Sequencing Kit SQK-LSK109 (Oxford Nanopore Technologies, Didkot, UK). Sequencing was performed using a MinION sequencer on a FLO-MIN114 flow cell (Oxford Nanopore Technologies) and the Native Barcoding Kit 24 V14 SQK-NBD114.24 (Oxford Nanopore Technologies) https://nanoporetech.com/document/ligation-sequencing-gdna-native-barcoding-v14-sqk-nbd114-24 (accessed on 10 April 2025). Basecalling was performed using MinKNOW 23.04.3, fast model configuration 400 bps.

*De novo* genome assembly was performed using Flye-2.9 https://github.com/mikolmogorov/Flye (accessed on 20 April 2025) with two iterations of the built-in polisher, followed by polishing using Medaka 2.1.1 software. Assembly completeness was assessed using BUSCO 5.8.0 in mode: prok_genome_prod, the lineage dataset was: corynebacteriales_odb10. Genome annotation was performed using the Bakta 1.9.4 https://github.com/oschwengers/bakta (accessed on 25 April 2025) with default settings. Species identification was performed based on average nucleotide identity (ANI) values calculated using the software pyani 0.2.12 https://github.com/asadprodhan/Average-Nucleotide-Identity-ANI-analysis (accessed on 30 April 2025) with ANIm method. The sequence was deposited in the GenBank database (BioProject PRJNA1257400, https://www.ncbi.nlm.nih.gov/bioproject/?term=PRJNA1257400, accessed on 15 March 2026).

The presence of plasmids was checked using Plasmidfinder 2.1.6 https://github.com/genomicepidemiology/plasmidfinder (accessed on 9 May 2025), GeNomad 1.12.0 https://github.com/apcamargo/genomad (accessed on 9 May 2025), and Integron_Finder 2.0.5 https://github.com/gem-pasteur/Integron_Finder (accessed on 9 May 2025). To predict antibacterial resistance, ncbi-amrfinderplus 3.12.8 https://github.com/ncbi/amr (accessed on 9 May 2025), Abricate https://github.com/tseemann/abricate (accessed on 9 May 2025), and staramr 0.12.1 https://github.com/phac-nml/staramr (accessed on 9 May 2025) tools were used. Clusters of biosynthetic pathways for biologically active compounds were identified using antiSMASH 6.1.1 https://antismash.secondarymetabolites.org/ (accessed on 9 May 2025). Genes relevant to the study were identified using the tblastn 2.12.0 algorithm https://blast.ncbi.nlm.nih.gov/ (accessed on 9 May 2025). Operon assignments were predicted using Operonmapper https://biocomputo.ibt.unam.mx/operon_mapper/ (accessed on 9 May 2025).

## 3. Results

### 3.1. Strain Isolation and Identification

A total of 315 strains were isolated from the perch (*P. fluviatilis*) skin. Analysis of QQ activity revealed that 32 isolates were capable of degrading AHL produced by *Aeromonas* sp. IR145. However, only six strains demonstrated persistent QQ activity and were selected for further study.

About 1500 bp sequences of all isolates were amplified and sequenced. The sequences were submitted to the GeneBank for accession numbers. The 16S ribosomal RNA sequences of PFS1.20, PFS1.69, PFS1.101, PFS2.95, PFS3.43 and PFS3.70 were assigned accession numbers as PRJNA1257400, PV596700.1, PV596701.1, PV596702.1, PV596703.1, and PV596704.1, respectively.

**Figure 2:**
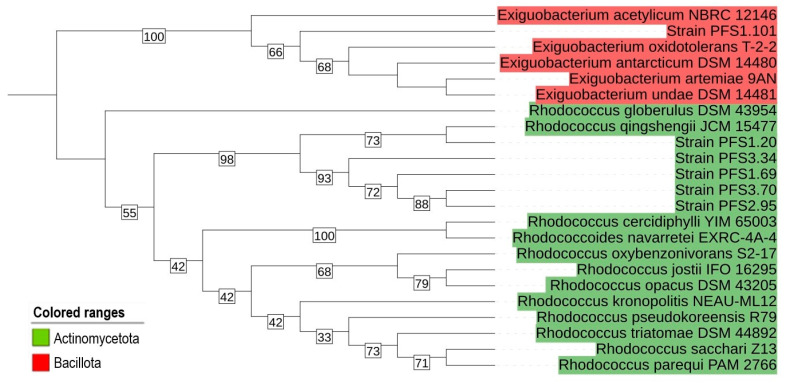
Phylogenetic tree resulting from nucleotide sequences of 16S rRNA genes of QQ isolates and the closest type strains from the NCBI database.

Phylogenetic analysis revealed that the chosen isolates belong to two phyla (Actinomycetota and Bacillota). Strains PFS1.20, PFS1.69, PFS2.95, PFS3.43, and PFS3.70 belong to the genus *Rhodococcus*, while strain PFS1.101 belongs to the genus *Exiguobacterium* (Figure 2).

### 3.2. Detection of qsdA and jydB Genesin Isolates

**Figure 3:**
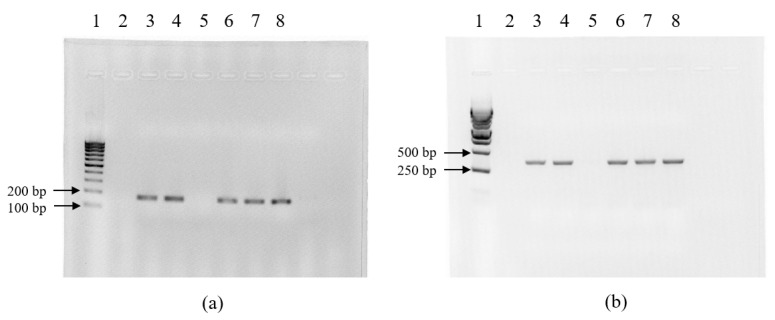
*jydB* (**a**) and *qsdA* (**b**) gene amplification products. 1—DNA length marker; 2—Negative control sample; 3—*Rhodococcus* sp. PFS1.20; 4—*Rhodococcus* sp. PFS1.69; 5—*Exiguobacterium* sp. PFS1.101; 6—*Rhodococcus* sp. PFS2.95; 7—*Rhodococcus* sp. PFS3.43; 8—*Rhodococcus* sp. PFS3.70.

PCR was used to screen for *qsdA* and *jydB* genes encoding AHL-degrading enzymes in isolates with confirmed QQ activity. These genes were detected in all isolates belonging to the genus *Rhodococcus*. The expected amplification product sizes were 300 bp and 168 bp for *qsdA* and *jydB*, respectively (Figure 3).

### 3.3. QQ Enzymatic Activity of Strains

**Figure 4:**
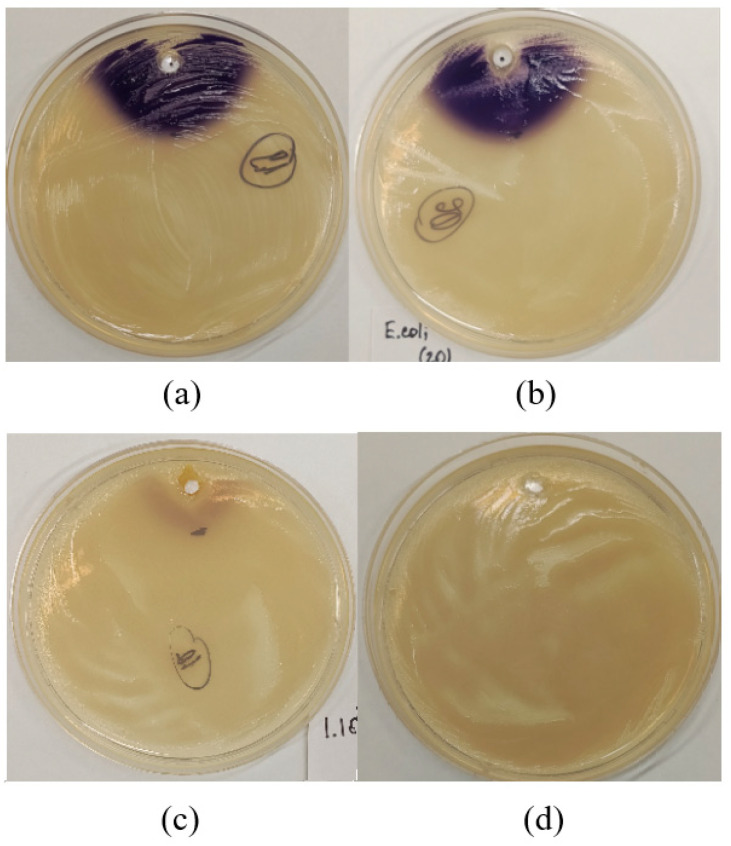
Destruction of AHL by selected isolates. (**a**) C6-AHL; (**b**) C6-AHL + *E. coli* BL21; (**c**) C6-AHL + *Exiguobacterium* sp. PFS 1.101; (**d**) C6-AHL + *Rhodococcus* sp. PFS1.20.

Quantitative assessment of the studied isolates for AHL degrading ability showed that *Rhodococcus* sp. PFS1.20, *Rhodococcus* sp. PFS1.69, *Rhodococcus* sp. PFS2.95, *Rhodococcus* sp. PFS3.43, *Rhodococcus* sp. PFS3.70 completely destroyed C6-AHL at a concentration of 10 μM within 24 h of cultivation. *Exiguobacterium* sp. PFS1.101 reduced the concentration of C6-AHL to 1 μM in the medium within the same time, complete degradation of AHL was observed after 48 h (Figure 4).

### 3.4. Enzymatic/Non-Enzymatic and Intra-/Extracellular QQ Activity

While assessing the enzymatic/non-enzymatic Quorum Quenching activity, a loss of the ability to inactivate AHL was observed after heating the cell cultures in all cases. These results indicate the enzymatic nature of QQ activity in the isolates.

The localization of QQ activity was also assessed. During the experiment, none of the cell-free culture fluids exhibited the ability to inactivate AHL, indicating the intracellular localization of QQ activity.

### 3.5. Evaluation of the Ability of QQ Strains to Inhibit the Growth and the Hemolytic Properties of Aeromonas

**Figure 5:**
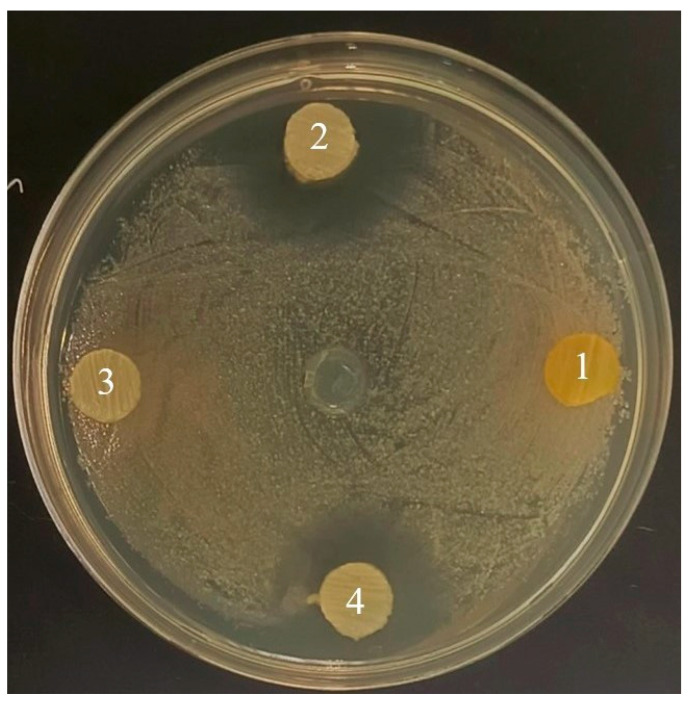
Inhibition of *A. salmonicida* A1 growth by QQ strains. 1—*Exiguobacterium* sp. PFS1.101; 2—*Rhodococcus* sp. PFS2.95; 3—*Rhodococcus* sp. PFS3.43; 4—*Rhodococcus* sp. PFS3.70.

The ability of the QQ isolates to inhibit the growth and hemolytic activity of *Aeromonas* bacteria was studied in vitro to evaluate their biotechnological potential. Strains *Rhodococcus* sp. PFS1.20, *Rhodococcus* sp. PFS2.95, and *Rhodococcus* sp. PFS3.70 actively inhibited the growth of *Aeromonas*. For *Rhodococcus* sp. PFS1.20, the diameters of inhibition zones in co-culture were 13 mm, 13 mm, 10 mm, and 8 mm with *A. salmonicida* W70, *A. salmonicida* A1, *A. bestiarum* VR410, and *A. piscicola* GPR23, respectively. For *Rhodococcus* sp. PFS2.95, the diameters of inhibition zones were 13 mm, 14 mm, 11 mm, and 10 mm with *A. salmonicida* W70, *A. salmonicida* A1, *A. bestiarum* VR410, and *A. piscicola* GPR23, respectively. For *Rhodococcus* sp. PFS3.70, the diameters of inhibition zones were 11 mm, 12 mm, 9 mm, and 9 mm for *A. salmonicida* W70, *A. salmonicida A1, A. bestiarum* VR410, and *A. piscicola* GPR23, respectively. Isolates *Rhodococcus* sp. PFS1.69, *Exiguobacterium* sp. PFS1.101 and *Rhodococcus* sp. PFS3.43 did not show growth inhibition of model *Aeromonas* strains (Table 1, Figure 5).

The test for suppression of *Aeromonas* hemolytic activity showed that strains *Rhodococcus* sp. PFS1.20, *Rhodococcus* sp. PFS1.69, *Rhodococcus* sp. PFS2.95, *Rhodococcus* sp. PFS3.43, and *Rhodococcus* sp. PFS3.70 were able to reduce the intensity of hemolysis when co-cultivated in 5% erythrocyte mass. *Exiguobacterium* sp. PFS1.101, in contrast, induced hemolysis, which makes it unsuitable for use as a probiotic (Table 2).

Based on the ability of the QQ strains to inhibit the growth and hemolytic activity of *Aeromonas* in vitro, *Rhodococcus* sp. PFS1.20, *Rhodococcus* sp. PFS2.95, and *Rhodococcus* sp. PFS3.70 were selected for further experiments.

### 3.6. Biofilm Suppression Ability of Bacteria of the Genus Rhodococcus

**Figure 6:**
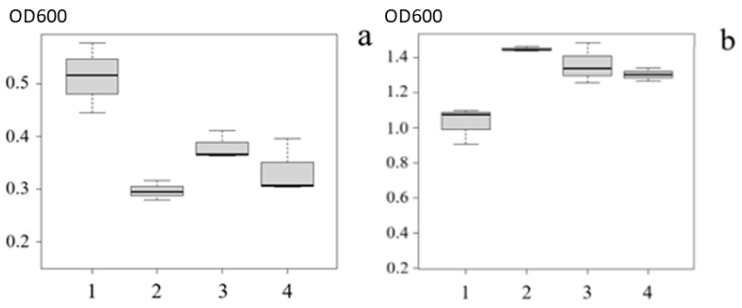
Optical density of biofilms (**a**) and planktonic cells (**b**) of the *A. salmonicida* A1 strain when exposed to lysates of the QQ isolates. 1—*A. salmonicida* A1 + *Rhodococcus* sp. PFS1.20; 2—*A. salmonicida* A1 + *Rhodococcus* sp. PFS2.95; 3—*A. salmonicida* A1 + *Rhodococcus* sp. PFS3.70; 4—*A. salmonicida* A1.

*Rhodococcus* sp. PFS1.20, *Rhodococcus* sp. PFS2.95, and *Rhodococcus* sp. PFS3.70 demonstrated the ability to inhibit biofilm formation in *A. salmonicida* A1. Biofilm growth was reduced by 44.4%, 27.3%, and 36.3%, respectively (Figure 6). Strains *A. salmonicida* W70, *A. piscicola* GPR23, and *A. bestiarum* VR410 formed virtually no biofilms, and therefore no significant inhibitory effect was observed. At the same time, the absence of a significant effect on the planktonic growth of aeromonads in liquid medium was noted.

### 3.7. Evaluation of the Ability of QQ Strains to Grow Under Different Environmental Factors

When using microorganisms as probiotics for aquaculture, it is necessary to evaluate their ability to survive under various conditions of the fish gastrointestinal tract, including different pH levels and the presence of bile acids. To simulate such conditions, we selected pH values of 3.0, 5.0, 7.0, and 9.0. It was found that the studied isolates could grow at all tested pH values. Moreover, the growth rate of the isolates in range of 5.0–9.0 pH was higher than at 3.0 pH. All measurements are presented in Table 3.

Evaluation of the isolates’ ability to grow under varying salinity showed that all strains were able to grow at NaCl concentrations ranging from 0% to 3%. The highest growth rate of the cultures was observed in a medium with 1% NaCl during the first 24 h of cultivation. Over the next 24 h, the growth rate decreased (Table S1).

Growth of the *Rhodococcus* sp. PFS1.20, *Rhodococcus* sp. PFS2.95 and *Rhodococcus* sp. PFS3.70 was reduced at all selected bile salt concentrations other than 0% (Table S2).

The growth capacity assessment showed that *Rhodococcus* sp. PFS1.20, *Rhodococcus* sp. PFS2.95, and *Rhodococcus* sp. PFS3.70 were capable of growing at temperatures ranging from 8 °C to 37 °C. The most active growth was observed at temperatures of 28 °C and 37 °C. At lower temperatures, growth slowed (Table 4).

### 3.8. Antimicrobial Resistance

The resistance of *Rhodococcus* sp. PFS1.20, *Rhodococcus* sp. PFS2.95, and *Rhodococcus* sp. PFS3.70 to AMD was assessed. The strains were found to be sensitive to all selected antibiotics.

### 3.9. Genome Sequencing and Assembly of Rhodococcus sp. PFS1.20

Based on the experimental results, *Rhodococcus* sp. PFS1.20 was selected as the most promising probiotic. The isolate’s genome was sequenced and annotated using ONT. Sequencing yielded 63,039 reads with an N50 of 15,917 bp. The longest read was 81,415 bp in length. Assembly yielded three contigs with a total length of 6,877,144 bp (N50 is 6,229,370 bp), the GC content was 62.4%, and the BUSCO assembly completeness was 92.1%.

**Figure 7:**
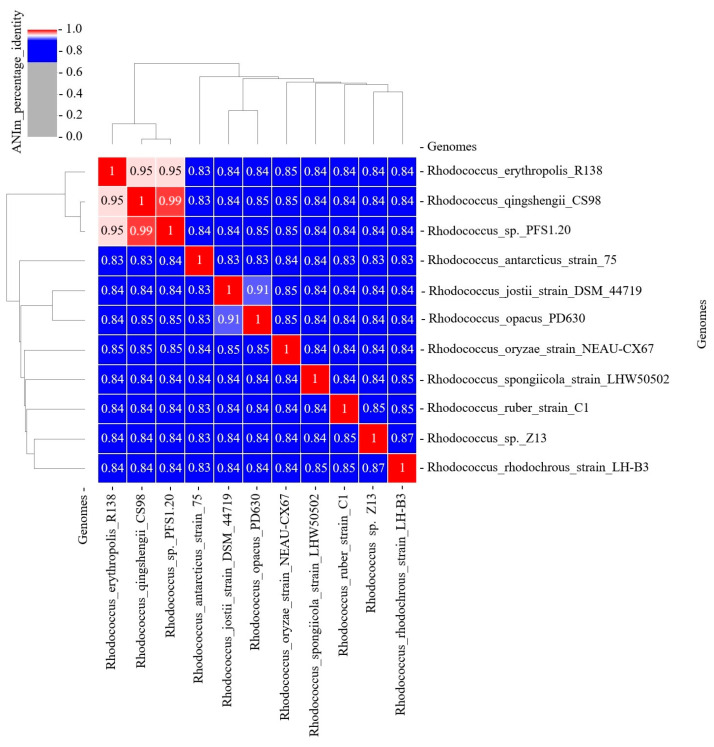
Average nucleotide identity of *Rhodococcus* sp. PFS1.20 and *Rhodococcus* type strains.

Whole-genome sequencing data were used to clarify the species affiliation of the isolate. *Rhodococcus* type strains genome sequences were obtained from the NCBI RefSeq database. Average nucleotide identity (ANI) values were calculated. The threshold for ANI is 95%, which is the basis for classifying a bacterium as a species. It was shown that the strain *Rhodococcus* sp. PFS1.20 exhibited a high (99%) ANI value relative to *R. qingshengii* CS98 (Figure 7). *R. qingshengii* was previously classified as a separate species but was later merged with *R. erythropolis* (ANI is 95%). Thus, the studied strain belongs to *R. erythropolis*.

Annotation of the obtained genome using Bakta v1.9.4 predicted 6810 protein-coding genes, 55 tRNA genes, 15 rRNA genes, 1 tmRNA gene, and 8 ncRNA genes. A search for plasmids and integrons did not reveal their presence in the genome of the studied strain.

Genes encoding QQ enzymes were detected in the genome. The *qsdA* gene for phosphotriesterase-like lactonase is located within the qsd operon. The *jydB* gene, encoding an α/β-hydrolase, is expressed independently. A number of genes promoting survival under oligotrophic conditions were identified. These include the isocitrate lyase and malate synthase genes, which play a role in the glyoxylate shunt; genes involved in the transformation of C1 compounds; and genes encoding ammonium transporters; genes of chaperones that maintain proteostasis under stressful conditions for bacteria (Table 5).

A number of gene clusters that contribute to the probiotic potential of the studied isolate were identified. These include genes encoding siderophores from the heterobactin and thermochelin groups, osmolytics, and carotenoids. Clusters encoding microbial antibiotics and bacteriocins, such as ε-Poly-L-lysine, xenematide, chloramphenicol, and corynecin III, were also identified. Furthermore, the AMRFinder tool identified genes encoding resistance factors to rifampin and chloramphenicol antibiotics (Table 6).

## 4. Discussion

Various bacteria use QS systems for density-dependent regulation of various gene expression, including virulence factors. Furthermore, microbial communities contain bacteria with QQ activity—the ability to degrade signaling molecules such as AHLs. In this study, AHL-degrading bacteria were isolated from the tissues of *P. fluviatilis*.

The proportion of QQ strains in the cultured perch microbiome was approximately 10%. These values are lower than published data. In the rainbow trout microbiome, 18% of isolates were capable of degrading C6-AHL [17]. In the microbiome of marine coelenterates, 19% of isolates were found capable of degrading C10-AHL [25]. Among them, only one-third were able to degrade both C10- and C6-AHLs. In the microbial communities of coastal ocean waters, 14% of microbiome demonstrated QQ activity [26], and in samples recovered from a depth of 2 km, their amount increases to 84% [27]. Such a low proportion of QQ isolates in perch may be associated with the specificity of QQ enzymes to AHLs with different side chain lengths. The strain used in this study as an AHL producer belongs to the genus *Aeromonas*, representatives of which produce C4-C6 AHL [28]. Therefore, strains possessing QQ enzymes that inactivate AHLs with long side chains remained undetected.

Most of the QQ isolates we identified belong to the genus *Rhodococcus*. Two QQ enzymes have been described for members of this genus to date. They belong to different families but cleave lactone compounds in a similar manner. The first and best-studied is the phosphotriesterase-like lactonase QsdA [29]. The second enzyme, the α/β-hydrolase JydB, was described relatively recently [30]. Using PCR, both genes were detected in all *Rhodococcus* isolates we studied. JydB exhibits high affinity for C4-HSL and 3-oxo-C6-HSL. The kcat/KM ratio for these compounds is 43 and 33 times higher than for C6-HSL [30]. QsdA degrades a wide range of lactones from C6 to C14 [29]. Thus, expression of two proteins at once likely allows bacteria to expand the spectrum of lactones they are able to hydrolyze.

Enzymatic QQ activity has not yet been described for microorganisms belonging to the genus *Exiguobacterium*. It is known that members of this genus are capable of synthesizing non-proteinaceous inhibitors of QS systems [31]. AHL degradation can occur not only enzymatically but also under the influence of environmental factors. Lactone rings are known to undergo reversible lactolysis at high pH and elevated temperatures. Furthermore, the stability of the AHL molecule is influenced by the length of the acyl chain: the longer the chain, the more stable the molecule [32].

QQ activity was not observed in cell-free supernatants of any of the strains studied. Furthermore, heating the suspension cultures also resulted in a loss of the ability to degrade AHL molecules. These data indicate that the QQ activity of the studied *Rhodococcus* isolates is due to the intracellular enzymes, which corresponds with literature data, as QsdA and JydB are intracellular enzymes [33,34]. Since enzymes with QQ activity have not been described in the literature for bacteria of the genus *Exiguobacterium*, our data may indicate the presence of previously unknown QQ enzymes or proteins with non-specific activity against AHLs.

Expression of genes encoding pathogenicity factors in *Aeromonas* is regulated by Quorum Sensing systems. It has been shown that disruption of QS leads to a significant reduction in pathogenicity [35]. In previous studies, *Bacillus*, *Kocuria*, and *Rhodococcus* inhibited the hemolytic properties of *Aeromonas* and suppressed biofilm formation [15,35,36]. Isolates *R. erythropolis* PFS1.20, *Rhodococcus* sp. PFS2.95, and *Rhodococcus* sp. PFS3.70 reduced the intensity of erythrocyte destruction by *Aeromonas*, indicating their potential use in the prevention of *Aeromonas* infections. Isolate *Exiguobacterium* PFS1.101 induced hemolysis of erythrocytes and was excluded from consideration as a potential probiotic. The probable cause of this phenomenon is synthesis of antibacterial compounds in sublethal concentrations by the *Exiguobacterium* PFS1.101 [37]. It has been shown that antibiotics in concentrations close to the minimum inhibitory concentration can stimulate expression of virulence factors in pathogenic microorganisms [38].

Isolates *R. erythropolis* PFS1.20, *Rhodococcus* sp. PFS2.95, and *Rhodococcus* sp. PFS3.70 actively inhibited the growth of *Aeromonas* on LB agar medium. These strains probably produce substances that have antagonistic activity against *Aeromonas*. Such cases have been described before [39,40]. At the same time, complete growth inhibition was not observed during planktonic growth in LB medium. A possible reason for this phenomenon is the synthesis of compounds with antibacterial properties at low concentrations by *Rhodococcus* isolates. It has been shown that subinhibitory concentrations of antibiotics can stimulate pathogen growth (hormesis) [41]. It should be noted that for *Aeromonas*, the phenomenon of growth stimulation by antibacterial drugs at low concentrations has not been studied sufficiently.

Microorganisms used as probiotics must be capable of growing in the gastrointestinal tract of the host organism. It is known that the pH level in the gastrointestinal tract of fish can vary from 2–4 in the stomach to 9–11 in the pyloric region with gradual neutralization in the intestine [36]. Therefore, it is necessary that probiotic candidates have the ability to grow under various conditions encountered in the fish gastrointestinal tract [14]. An alternative approach is the direct delivery of purified enzymes, but this requires ensuring that the enzyme remains active in the gastrointestinal tract [12]. Strains of *R. erythropolis* PFS1.20, *Rhodococcus* sp. PFS2.95, and *Rhodococcus* sp. PFS3.70 demonstrated the ability to grow in a medium with a pH of 3–9, indicating their probiotic potential. The observed tolerance of the strains to various pH levels is characteristic of the genus *Rhodococcus* as a whole, which, like many other Actinomycetota is characterized by high adaptability to environmental conditions [42].

Efficient fish farming in aquaculture requires maintaining optimal conditions such as water temperature and salinity. For the most common fish species in aquaculture (e.g., rainbow trout and carp), optimal water temperatures are in the range of 12–18 °C [43]. *R. erythropolis* PFS1.20, *Rhodococcus* sp. PFS2.95, and *Rhodococcus* sp. PFS3.70 demonstrated the ability to grow over a wide temperature span (from 8 °C to 37 °C). Furthermore, isolates retained their ability to grow in salinity conditions ranging from 0% to 3%. These results suggest the potential use of these strains for infection prevention in both freshwater and marine fish aquaculture.

The resistance of orally administered probiotic strains to bile acids is an important criterion for their selection. In our study, the strains *R. erythropolis* PFS1.20, *Rhodococcus* sp. PFS2.95, and *Rhodococcus* sp. PFS3.70 ceased growth in the presence of bile salts, which severely limits the potential for their application. A possible solution to this problem is the protection of probiotic cells via microencapsulation. Encapsulation allows the probiotic to be shielded from the effects of gastric juice and the bile salts of animals [44]. For instance, Gunzburg W.H. and co-authors demonstrated that the encapsulation of probiotic bacteria and yeast with cellulose sulfate significantly increased the resistance of microorganisms to the conditions of the murine gastrointestinal tract [45]. Encapsulation of the *Lactococcus lactis* A12 strain, used as a probiotic for fish, enhanced its viability and stability compared to the spray-dried strain [46]. It should be noted that the implementation of this approach is associated with a number of challenges, including the need to select safe and cost-effective components for strain encapsulation [47].

Uncontrolled usage of AMD can lead to the spread of resistance in the microbial community, for example, through horizontal gene transfer [48]. Resistance to AMD in probiotic strains is highly undesirable. In our study, all strains were sensitive to the antibiotics used.

Genomic analysis of the *R. erythropolis* PFS1.20 revealed a wide range of genetic determinants that provide its adaptive potential and competitive advantages in various ecological niches. Its antagonistic properties against pathogenic Aeromonas can be explained by a combination of several factors: the ability to inactivate QS signaling molecules, the production of antimicrobial compounds (ε-polylysine, xenematide, chloramphenicol, and corynecin III), and competition for iron through the production of siderophores.

Genome of the isolate *R. erythropolis* PFS1.20 contains genes encoding QQ enzymes: *qsdA* and *jydB* [30,49]. Inactivation of QS signaling molecules may provide competitive advantages by disrupting communication between pathogenic bacteria and suppressing expression of their virulence factors. *Aeromonas* predominantly produce C4-C6 AHLs [28], which are actively degraded by these enzymes [29,30].

Biosynthetic clusters for various antimicrobial compounds were found in the genome of the studied isolate. Among them is the biosynthesis cluster of ε-poly-L-lysine, a cationic antimicrobial peptide whose mechanism of action involves disrupting the integrity and permeability of the cell walls and membranes of bacteria and fungi, as well as inducing the accumulation of reactive oxygen species inside the cell [50]. A biosynthesis cluster of xenematide, a cyclic peptide antibiotic, was also found [51], as well as biosynthesis clusters of chloramphenicol and corynecin III [52]. The action mechanism of corynecin III is to inhibit protein synthesis by affecting the bacterial ribosome, which disrupts the translation process. Antibacterial resistance genes were identified in the genome: the rifampin monooxygenase *iri* gene and the *cmrA* transporter protein gene, which mediates the efflux of chloramphenicol. The presence of chloramphenicol-related biosynthetic clusters in the genome suggests the presence of resistance mechanisms to such AMD. It should be noted that the programs ABRicate, staramr, and AMRFinder were used to search for determinants of antibacterial resistance; however, only the latter identified the aforementioned genes, which may indicate the low reliability of results obtained using certain bioinformatics tools and the necessity of their further experimental validation. The susceptibility of the studied strain to rifampin has been demonstrated experimentally. The discrepancy between phenotypic properties predicted from genomic data and the results of microbiological tests is a well-known issue, attributable both to the imperfections of gene annotation algorithms and the possible presence of biochemical shunts, as well as the likely reduction in gene expression due to mutations whose effects are difficult to predict.

In the genome of the studied strain, a biosynthetic gene cluster for heterobactin, a siderophore specific to the genus *Rhodococcus*, was identified [53]. The production of siderophores ensures the efficient chelation of iron ions in the environment, making them unavailable to competing microorganisms.

For probiotic microorganisms used in aquaculture, a crucial adaptive trait is their ability to survive under stressful conditions, such as elevated salinity, temperature fluctuations, and nutrient deficiency.

Chaperones play a key role in maintaining cellular homeostasis under stress by ensuring proper protein folding and preventing their aggregation. In the genome of *R. erythropolis* PFS1.20, genes encoding the molecular chaperones DnaK and GroEL, as well as the ATP-dependent chaperone ClpB, were detected, indicating the presence of a developed proteostasis system [54].

The genome of *R. erythropolis* PFS1.20 was also found to contain a biosynthetic gene cluster for ectoine, an osmolyte characteristic of halophilic and halotolerant prokaryotes that protects cells from osmotic stress [55]. Furthermore, a gene encoding the ProV permease protein, a component of the ABC transport system for L-proline and glycine betaine uptake, was identified [56]. These compounds also function as osmoprotectants, accumulating in the cytoplasm and maintaining cell turgor pressure under hyperosmotic stress. The presence of these genetic determinants endows strain *R. erythropolis* PFS1.20 with resistance to salinity fluctuations, which represents a significant advantage for its application in marine aquaculture. An additional factor enhancing the isolate’s survival in the environment is the presence of a biosynthetic gene cluster for carotenoids, notably phytoene, which acts as a photoprotectant, shielding cells from ultraviolet radiation damage [57]. 

The ability to grow under nutrient-limited conditions has been extensively documented for the genus *Rhodococcus*. The growth of *R. erythropolis* N9T-4 on solid mineral medium without additional carbon or energy sources has been described [58]. Several studies have reported increased expression of genes associated with aldehyde metabolism, as well as the glyoxylate and other shunts, under oligotrophic conditions [59,60]. The genome of the studied isolate, *R. erythropolis* PFS1.20, contains a number of genes facilitating adaptation to oligotrophy. These include, for instance, the gene for isocitrate lyase, involved in the glyoxylate shunt – an alternative pathway of the tricarboxylic acid cycle that enables the utilization of two-carbon compounds as carbon and energy sources [61]. Functionally linked to it, the gene for malate synthase G was also detected. Furthermore, genes involved in the transformation of inorganic carbon compounds were identified, including the gene for the large subunit of carbon monoxide dehydrogenase, suggesting the potential ability of the strain to utilize CO as a possible energy source. An additional mechanism contributing to survival under resource limitation is the presence of the *aldA* gene encoding aldehyde dehydrogenase, involved in the metabolism of aldehydes, which may also serve as alternative carbon sources. To ensure nitrogen supply under oligotrophic conditions, the ammonium transporter gene *amtB* is present, enabling efficient scavenging of trace amounts of ammonium from the environment [54,62].

In conclusion, genomic analysis of the isolate *R. erythropolis* PFS1.20 revealed a suite of genetic determinants underpinning its broad metabolic potential, resistance to stress factors, and antagonistic activity against other microorganisms.

## 5. Conclusions

In this work, we successfully isolated six novel bacterial strains that are active degraders of AHL. The isolates belong to the genera *Rhodococcus* and *Exiguobacterium*. Investigation of the quorum quenching (QQ) properties of the strains demonstrated that the AHL-degrading activity is enzymatic in nature and is localized intracellularly. This study expands the known diversity of organisms possessing QQ activity. Furthermore, the genome of the strain *R. erythropolis* PFS1.20 was sequenced and analyzed. This strain inhibits the growth and pathogenic properties of bacteria from the genus *Aeromonas* and grows under conditions of varying salinity, pH, and temperature. Growth inhibition of the strain by bile salts limits the scope of its application and necessitates the development of protective measures against bile exposure, such as encapsulation. Nevertheless, this strain possesses high probiotic potential and could be used for the development of agents for the prevention of aeromonosis in aquaculture following in vivo trials on economically important fish species, such as *Salmo salar*, *Carassius gibelio*, and *Oncorhynchus mykiss*.

## Figures and Tables

**Table 1:** Inhibition of *Aeromonas* bacterial growth by QQ isolates (diameter of inhibition zones, mm).

	*A. salmonicida* W70	*A. salmonicida* A1	*A. bestiarum* VR410	*A. piscicola* GPR23
*Rhodococcus* sp. PFS1.20	13 mm	13 mm	10 mm	8 mm
*Rhodococcus* sp. PFS1.69	0	0	0	0
*Exiguobacterium* sp. PFS1.101	0	0	0	0
*Rhodococcus* sp. PFS2.95	13 mm	14 mm	11 mm	10 mm
*Rhodococcus* sp. PFS3.43	0	0	0	3 mm
*Rhodococcus* sp. PFS3.70	11 mm	12 mm	9 mm	9 mm

**Table 2:** Suppression of hemolytic properties of *Aeromonas* bacteria by strains possessing Quorum Quenching properties.

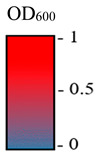	LB	*A. salmonicida* A1	*A. salmonicida* W70	*A. piscicola* GPR23	*A. bestiarum* VR410
LB	0	0.313	0.043	0.096	0.193
*Rhodococcus* sp. PFS1.20	−0.002	0.430	−0.089	−0.107	0.121
*Rhodococcus* sp. PFS1.69	−0.101	0.498	−0.037	−0.107	0.077
*Exiguobacterium* sp. PFS1.101	−0.054	0.348	0.491	0.240	0.324
*Rhodococcus* sp. PFS2.95	−0.101	0.338	−0.014	−0.106	0.119
*Rhodococcus* sp. PFS3.43	0.017	0.240	0.013	0.015	0.121
*Rhodococcus* sp. PFS3.70	−0.106	0.201	−0.012	−0.077	0.211

**Table 3:** Growth of *Rhodococcus* sp. PFS1.20, *Rhodococcus* sp. PFS2.95, and *Rhodococcus* sp. PFS3.70 isolates under different pH levels.

Isolate	pH	Day 0 (OD_600_)	Day 1 (OD_600_)	Growth Rate, h^−1^
*Rhodococcus* sp. PFS1.20	pH = 3.0	0.085	0.113	0.012
	pH = 5.0	0.1	0.499	0.067
	pH = 7.0	0.097	0.485	0.067
	pH = 9.0	0.109	0.417	0.056
*Rhodococcus* sp. PFS2.95	pH = 3.0	0.068	0.129	0.027
	pH = 5.0	0.1	0.499	0.067
	pH = 7.0	0.087	0.417	0.065
	pH = 9.0	0.09	0.338	0.055
*Rhodococcus* sp. PFS3.70	pH = 3.0	0.058	0.074	0.010
	pH = 5.0	0.07	0.384	0.071
	pH = 7.0	0.097	0.417	0.061
	pH = 9.0	0.061	0.338	0.071

**Table 4:** Growth of *Rhodococcus* sp. PFS1.20, *Rhodococcus* sp. PFS2.95, and *Rhodococcus* sp. PFS3.70 under different temperature conditions.

Isolates	Temperature	Day 0 (OD_600_)	Day 1 (OD_600_)	Specific Growth Rate, h^−1^	Day 2 (OD_600_)	Specific Growth Rate, h^−1^
*Rhodococcus* sp. PFS1.20	8	0.078	0.169	0.032	0.254	0.017
	15	0.118	0.182	0.018	0.226	0.009
	28	0.075	0.526	0.081	0.747	0.015
	37	0.075	0.649	0.090	0.747	0.006
*Rhodococcus* sp. PFS2.95	8	0.075	0.156	0.031	0.254	0.020
	15	0.118	0.148	0.009	0.226	0.018
	28	0.074	0.285	0.056	0.747	0.040
	37	0.074	0.201	0.042	0.84	0.060
*Rhodococcus* sp. PFS3.70	8	0.075	0.152	0.029	0.254	0.021
	15	0.104	0.182	0.023	0.226	0.009
	28	0.075	0.239	0.048	0.497	0.031
	37	0.078	0.169	0.032	0.497	0.045

**Table 5:** Genes that determine probiotic potential, identified in the genome of *R. erythropolis* PFS1.20.

Gene (Accession Number)	Contig	Coordinates	Identity, %
isocitrate lyase (WP_043803320.1)	1	1,163,198–1,164,487	93
malate synthase G (WP_064074336.1)	1	2,686,544–2,688,727	100
carbon-monoxide dehydrogenase large subunit (WP_042448026.1)	1	5,581,743–5,584,265	99.8
aldehyde dehydrogenase AldA (WP_017681519.1)	1	5,150,580–5,152,106	64.9
ammonium transporter AmtB (BAH33147.1)	1	2,042,660–2,043,919	88.5
molecular chaperone DnaK (WP_019749384.1)	1	935,305–937,158	99.8
molecular chaperone GroEL (WP_003940715.1)	1	1,559,014–1,560,624	100
ATP-dependent chaperone ClpB (WP_003944582.1)	1	978,176–980,729	100
L-proline glycine betaine ABC transport system permease protein ProV (QHE71607.1)	1	4,572,754–4,573,748	83.98
*qsdA* (AGA94625.1)	1	5,042,560–5,043,531	99
*jydB* (WP_230359177.1)	1	5,199,513–5,200,319	100
rifampin monooxygenase Iri (WP_063851318.1)	1	441,957–443,129	95.56
chloramphenicol efflux MFS transporter CmrA (WP_063844243.1)	2	441,957–443,129	99.74

**Table 6:** Biosynthesis clusters, found in *R. erythropolis* PFS1.20 genome.

Biosynthesis Clusters	Contig	Coordinates
thermochelin	1	375,585–433,776
heterobactin B	1	2,117,857–2,168,548
carotenoid phytoene	1	3,041,601–3,066,966
ectoine	1	3,265,869–3,276,267
ε-Poly-L-lysine	1	3524–37,417
corynecin III	1	1,309,512–1,417,351
xenematide	2	15,661–49,670
chloramphenicol	2	411,475–470,142

## Data Availability

The original contributions presented in this study are included in the article/Supplementary Materials. Further inquiries can be directed to the corresponding authors.

## Supplementary Materials

The following supporting information can be downloaded at: https://www.mdpi.com/article/10.3390/ani16091339/s1, Table S1: Growth of *Rhodococcus* sp. PFS1.20, *Rhodococcus* sp. PFS2.95, and *Rhodococcus* sp. PFS3.70 under varying salinity; Table S2: Growth of *Rhodococcus* sp. PFS1.20, *Rhodococcus* sp. PFS2.95, and *Rhodococcus* sp. PFS3.70 strains in the presence of bile salts.

## References

[1] Ampomah-Wireko M., Luo C., Cao Y., Wang H., Nininahazwe L., Wu C. (2021). Chemical probe of AHL modulators on quorum sensing in Gram-Negative Bacteria and as antiproliferative agents: A review. Eur. J. Med. Chem..

[2] Zaytseva Y.V., Sidorov A.V., Marakaev O.A., Khmel I.A. (2019). Plant-Microbial Interactions Involving Quorum Sensing Regulation. Microbiology.

[3] Bartie K.L., Desbois A.P. (2024). *Aeromonas dhakensis*: A Zoonotic Bacterium of Increasing Importance in Aquaculture. Pathogens.

[4] Dong Y.H., Wang L.Y., Zhang L.H. (2007). Quorum-quenching microbial infections: Mechanisms and implications. Philos. Trans. R. Soc. Lond. B Biol. Sci..

[5] Essock-Burns T., Bennett B.D., Arencibia D., Moriano-Gutierrez S., Medeiros M., McFall-Ngai M.J., Ruby E.G. (2021). Bacterial Quorum-Sensing Regulation Induces Morphological Change in a Key Host Tduring the *Euprymna scolopes–Vibrio fischeri* Symbiosis. mBio.

[6] Soh E.Y., Smith F., Gimenez M.R., Yang L., Vejborg R.M., Fletcher M., Halliday N., Bleves S., Heeb S., Cámara M. (2021). Disruption of the *Pseudomonas aeruginosa* Tat system perturbs PQS-dependent quorum sensing and biofilm maturation through lack of the Rieske cytochrome *bc*1 sub-unit. PLoS Pathog..

[7] Uroz S., Dessaux Y., Oger P. (2009). Quorum sensing and quorum quenching: The yin and yang of bacterial communication. ChemBioChem.

[8] Chen F., Gao Y., Chen X., Yu Z., Li X. (2013). Quorum quenching enzymes and their application in degrading signal molecules to block quorum sensing-dependent infection. Int. J. Mol. Sci..

[9] Kusada H., Zhang Y., Tamaki H., Kimura N., Kamagata Y. (2019). Novel *N*-Acyl Homoserine Lactone-Degrading Bacteria Isolated From Penicillin-Contaminated Environments and Their Quorum-Quenching Activities. Front. Microbiol..

[10] Zhang B., Zhuang X., Guo L., McLean R.J.C., Chu W. (2019). Recombinant N-acyl homoserine lactone-Lactonase AiiA_QSI-1_ Attenuates *Aeromonas hydrophila* Virulence Factors, Biofilm Formation and Reduces Mortality in Crucian Carp. Mar. Drugs..

[11] Peng M., Tong W., Zhao Z., Xiao L., Wang Z., Liu X., He X., Song Z. (2021). Attenuation of *Aeromonas hydrophila* Infection in *Carassius auratus* by YtnP, a *N*-acyl Homoserine Lactonase from *Bacillus licheniformis* T-1. Antibiotics.

[12] Sun X., Liu J., Yan Y., Yang S., Zhang G., Mohamed H.F. (2025). Development and Biochemical Characterization of Quorum Quenching Enzyme from Deep-Sea *Bacillus velezensis* DH82. Microorganisms.

[13] Shaheer P., Sreejith V.N., Joseph T.C., Murugadas V., Lalitha K.V. (2021). Quorum quenching *Bacillus* spp.: An alternative biocontrol agent for *Vibrio harveyi* infection in aquaculture. Dis. Aquat. Organ..

[14] James G., Radhakrishnan V., Marathippallam Jamal J., Prasannan Geetha P., Pillai D., Vattiringal Jayadradhan R.K. (2025). Exploring the probiotic potential of *B.cereus* and *B. velezensis* associated with mangrove sediments in aquaculture. Discov. Ocean..

[15] Chen Y., Rong Y., Wang R., Guo Z., Chi Z., Xu Z., Prazdnova E.V., Chikindas M.L., St-Hilaire S., Kim T.-J. (2026). Aquaculture pond is a rich source of Streptomyces spp. with promising potential for developing quorum sensing manipulating probiotics. Aquac. Rep..

[16] McClean K.H., Winson M.K., Fish L., Taylor A., Chhabra S.R., Camara M., Daykin M., Lamb J.H., Swift S., Bycroft B.W. (1997). Quorum sensing and *Chromobacterium violaceum*: Exploitation of violacein production and inhibition for the detection of *N*-acylhomoserine lactones. Microbiology.

[17] Torabi Delshad S., Soltanian S., Sharifiyazdi H., Haghkhah M., Bossier P. (2018). Identification of *N*-acyl homoserine lactone-degrading bacteria isolated from rainbow trout (*Oncorhynchus mykiss*). J. Appl. Microbiol..

[18] Stecher G., Suleski M., Tao Q., Tamura K., Kumar S. (2025). MEGA 12.1: Cross-Platform Release for macOS and Linux Operating Systems. J. Mol. Evol..

[19] Khmel I.A., Sorokina T.A., Lipasova V.A., Metlitskaya A.Z., Antonenko A.M., Chernin L.S. (1998). Biological control of crown gall in grapevine and raspberry by two *Pseudomonas* spp. with a wide spectrum of antagonistic activity. Biocontrol Sci. Technol..

[20] Zhao L., Jin X., Xiong Z., Tang H., Guo H., Ye G., Chen D., Yang S., Yin Z., Fu H. (2022). The Antivirulence Activity of Umbelliferone and Its Protective Effect against *A. hydrophila*-Infected Grass Carp. Int. J. Mol. Sci..

[21] Sun B., Luo H., Jiang H., Wang Z., Jia A. (2021). Inhibition of Quorum Sensing and Biofilm Formation of Esculetin on *Aeromonas hydrophila*. Front. Microbiol..

[22] Zaitseva J., Granik V., Belik A., Koksharova O., Khmel I. (2009). Effect of nitrofurans and NO generators on biofilm formation by Pseudomonas aeruginosa PAO1 and Burkholderia cenocepacia 370. Res. Microbiol..

[23] Trenkenshu R.P. (2019). Calculation of the specific growth rate of microalgae. Mar. Biol. J..

[24] Biemer J.J. (1973). Antimicrobial susceptibility testing by the Kirby-Bauer disc diffusion method. Ann. Clin. Lab. Sci..

[25] Reina J.C., Torres M., Llamas I. (2019). *Stenotrophomonas maltophilia* AHL-Degrading Strains Isolated from Marine Invertebrate Microbiota Attenuate the Virulence of *Pectobacterium carotovorum* and *Vibrio coralliilyticus*. Mar. Biotechnol..

[26] Romero M., Martin-Cuadrado A.B., Roca-Rivada A., Cabello A.M., Otero A. (2011). Quorum quenching in cultivable bacteria from dense marine coastal microbial communities. FEMS Microbiol. Ecol..

[27] Muras A., López-Pérez M., Mayer C., Parga A., Amaro-Blanco J., Otero A. (2018). High Prevalence of Quorum-Sensing and Quorum-Quenching Activity among Cultivable Bacteria and Metagenomic Sequences in the Mediterranean Sea. Genes.

[28] Chan K.G., Puthucheary S.D., Chan X.Y., Yin W.F., Wong C.S., See Too W.S., Chua K.H. (2011). Quorum Sensing in *Aeromonas* Species Isolated from Patients in Malaysia. Curr. Microbiol..

[29] Uroz S., Oger P.M., Chapelle E., Adeline M.T., Faure D., Dessaux Y. (2008). A *Rhodococcus qsdA*-encoded enzyme defines a novel class of large-spectrum quorum-quenching lactonases. Appl. Environ. Microbiol..

[30] Ryu D.H., Lee S.W., Mikolaityte V., Kim Y.W., Jeong H.Y., Lee S.J., Lee C.H., Lee J.K. (2020). Identification of a Second Type of AHL-lactonase from *Rhodococcus* sp. BH4, belonging to the α/β Hydrolase Superfamily. J. Microbiol. Biotechnol..

[31] Singh V.K., Mishra A., Jha B. (2019). 3-Benzyl-Hexahydro-Pyrrolo [1,2-a]Pyrazine-1,4-Dione Extracted From *Exiguobacterium indicum* Showed Anti-biofilm Activity Against *Pseudomonas aeruginosa* by Attenuating Quorum Sensing. Front. Microbiol..

[32] Yates E.A., Philipp B., Buckley C., Atkinson S., Chhabra S.R., Sockett R.E., Goldner M., Dessaux Y., Cámara M., Smith H. (2002). *N*-Acylhomoserine lactones undergo lactonolysis in a pH-, temperature-, and acyl chain length-dependent manner during growth of *Yersinia pseudotuberculosis* and *Pseudomonas aeruginosa*. Infect. Immun..

[33] Czajkowski R., Jafra S. (2009). Quenching of acyl-homoserine lactone-dependent quorum sensing by enzymatic disruption of signal molecules. Acta Biochim. Pol..

[34] Christiaen S.E., Brackman G., Nelis H.J., Coenye T. (2011). Isolation and identification of quorum quenching bacteria from environmental samples. J. Microbiol. Methods.

[35] Chu W., Zhou S., Zhu W., Zhuang X. (2014). Quorum quenching bacteria *Bacillus* sp. QSI-1 protect zebrafish (*Danio rerio*) from *Aeromonas hydrophila* infection. Sci. Rep..

[36] Sharifuzzaman S., Rahman H., Austin D.A., Austin B. (2018). Properties of Probiotics *Kocuria* SM1 and *Rhodococcus* SM2 Isolated from Fish Guts. Probiotics Antimicrob. Proteins.

[37] Cavanaugh N.T., Parthasarathy A., Wong N.H., Steiner K.K., Chu J., Adjei J., Hudson A.O. (2021). *Exiguobacterium* sp. is endowed with antibiotic properties against Gram positive and negative bacteria. BMC Res. Notes.

[38] Vasilchenko A.S., Rogozhin E.A. (2019). Sub-inhibitory Effects of Antimicrobial Peptides. Front. Microbiol..

[39] Kitagawa W., Hata M. (2022). Complete Genome Sequence of *Rhodococcus erythropolis* JCM 2895, an Antibiotic Protein-Producing Strain. Microbiol. Resour. Announc..

[40] Harunari E., Bando M., Igarashi Y. (2022). Rausuquinone, a non-glycosylated pluramycin-class antibiotic from *Rhodococcus*. J. Antibiot..

[41] Gorr S.U., Brigman H.V., Anderson J.C., Hirsch E.B. (2022). The antimicrobial peptide DGL13K is active against drug-resistant gram-negative bacteria and sub-inhibitory concentrations stimulate bacterial growth without causing resistance. PLoS ONE.

[42] Iminova L., Delegan Y., Frantsuzova E., Bogun A., Zvonarev A., Suzina N., Anbumani S., Solyanikova I. (2022). Physiological and biochemical characterization and genome analysis of *Rhodococcus qingshengii* strain 7B capable of crude oil degradation and plant stimulation. Biotechnol. Rep..

[43] Jiang X., Dong S., Liu R., Huang M., Dong K., Ge J., Gao Q., Zhou Y. (2021). Effects of temperature, dissolved oxygen, and their interaction on the growth performance and condition of rainbow trout (*Oncorhynchus mykiss*). J. Therm. Biol..

[44] Vijayaram S., Sinha R., Faggio C., Ringø E., Chou C.C. (2024). Biopolymer encapsulation for improved probiotic delivery: Advancements and challenges. AIMS Microbiol..

[45] Gunzburg W.H., Aung M.M., Toa P., Ng S., Read E., Tan W.J., Brandtner E.M., Dangerfield J., Salmons B. (2020). Efficient protection of microorganisms for delivery to the intestinal tract by cellulose sulphate encapsulation. Microb. Cell Factories.

[46] Valle Vargas M.F., Ruiz Pardo R.Y., Villamil-Díaz L., Alean J., Santagapita P.R., Quintanilla-Carvajal M.X. (2025). Encapsulation improves viability and stability of spray-dried *Lactococcus lactis* A12 for inclusion in fish feed. PLoS ONE.

[47] Zhu Y., Lv L., Du B., Zhao M. (2026). Next-Generation Microencapsulation Technologies for Probiotic Protection and Precision Delivery. Microb. Biotechnol..

[48] Fang L., Chen R., Li C., Sun J., Liu R., Shen Y., Guo X. (2024). The association between the genetic structures of commonly incompatible plasmids in Gram-negative bacteria, their distribution and the resistance genes. Front. Cell Infect. Microbiol..

[49] Chane A., Barbey C., Bourigault Y., Maillot O., Rodrigues S., Bouteiller M., Merieau A., Konto-Ghiorghi Y., Beury-Cirou A., Guespin-Michel J. (2019). A flavor lactone mimicking AHL quorum-sensing signals exploits the broad affinity of the QsdR regulator to stimulate transcription of the rhodococcal qsd operon involved in quorum-quenching and biocontrol activities. Front. Microbiol..

[50] Wang L., Zhang C., Zhang J., Rao Z., Xu X., Mao Z., Chen X. (2021). Epsilon-poly-L-lysine: Recent advances in biomanufacturing and applications. Front. Bioeng. Biotechnol..

[51] Hung K.Y., Harris P.W., Heapy A.M., Brimble M.A. (2011). Synthesis and assignment of stereochemistry of the antibacterial cyclic peptide xenematide. Org. Biomol. Chem..

[52] Nakano H., Tomita F., Yamaguchi K., Nagashima M., Suzuki T. (1977). Corynecin (chloramphenicol analogs) fermentation studies: Selective production of Corynecin I by Corynebacterium hydrocarboclastus grown on acetate. Biotechnol. Bioeng..

[53] Bosello M., Zeyadi M., Kraas F.I., Linne U., Xie X., Marahiel M.A. (2013). Structural characterization of the heterobactin siderophores from Rhodococcus erythropolis PR4 and elucidation of their biosynthetic machinery. J. Nat. Prod..

[54] Gallegos A.L., Nashmias M.E., Zubimendi J.P., Hernández M.A., Acosta V., Tejerizo G.A.T., Alvarez H.M. (2025). Adaptive responses of *Rhodococcus aetherivorans* L13 to oligotrophy: Genome and transcriptomic analysis. Curr. Genet..

[55] Liu M., Liu H., Shi M., Jiang M., Li L., Zheng Y. (2021). Microbial production of ectoine and hydroxyectoine as high-value chemicals. Microb. Cell Factories.

[56] Heo S., Lee J., Lee J.H., Jeong D.W. (2019). Genomic Insight into the Salt Tolerance of *Enterococcus faecium*, *Enterococcus faecalis* and *Tetragenococcus halophilus*. J. Microbiol. Biotechnol..

[57] Jiang W., Sun J., Gao H., Tang Y., Wang C., Jiang Y., Zhang W., Xin F., Jiang M. (2023). Carotenoids production and genome analysis of a novel carotenoid producing *Rhodococcus aetherivorans* N1. Enzym. Microb. Technol..

[58] Ohhata N., Yoshida N., Egami H., Katsuragi T., Tani Y., Takagi H. (2007). An extremely oligotrophic bacterium, *Rhodococcus erythropolis* N9T-4, isolated from crude oil. J. Bacteriol..

[59] Yoshida N., Hayasaki T., Takagi H. (2011). Gene expression analysis of Methylotrophic oxidoreductases involved in the oligotrophic growth of *Rhodococcus erythropolis* N9T-4. Biosci. Biotechnol. Biochem..

[60] Yano T., Yoshida N., Yu F., Wakamatsu M., Takagi H. (2015). The glyoxylate shunt is essential for CO2-requiring oligotrophic growth of *Rhodococcus erythropolis* N9T-4. Appl. Microbiol. Biotechnol..

[61] Yano T., Funamizu Y., Yoshida N. (2016). Intracellular accumulation of Trehalose and glycogen in an extreme oligotroph, Rhodococcus erythropolis N9T-4. Biosci. Biotechnol. Biochem..

[62] Yoshida N., Inaba S., Takagi H. (2014). Utilization of atmospheric ammonia by an extremely oligotrophic bacterium, Rhodococcus erythropolis N9T-4. J. Biosci. Bioeng..

