# Supplementary material for: Biotechnological Potential of Quorum Quenching Bacterial Strains Isolated from Perca fluviatilis
Source: Animals (Basel). 2026 Apr 27;16(9):1339. doi: 10.3390/ani16091339 (PMC13163003; doi:10.3390/ani16091339)
Supplement: Supplementary file 1 [file animals-16-01339-s001.zip › animals-4250242-supplementary.pdf]

Table S1. Growth of *Rhodococcus* sp. PFS1.20, *Rhodococcus* sp. PFS2.95, and *Rhodococcus* sp. PFS3.70 under varying salinity.

Isolate	NaCl concentration, %	Starting conditions		Day 1			Day 2		
		CFU	OD600	CFU	OD600	Specific growth rate, h^{-1}	CFU	OD600	Specific growth rate, h^{-1}
Rhodococcus sp. PFS1.20	0	2×10^6	0.118	7×10^7	0.649	0.147	2.17×10^8	1.101	0.045
	0.5		0.116	8×10^7	0.633	0.152	3.17×10^8	1.13	0.056
	1		0.132	1×10^8	0.656	0.164	2×10^8	0.921	0.024
	2		0.142	2×10^8	0.677	0.185	3.17×10^8	0.881	0.022
	3		0.117	6×10^7	0.596	0.141	7×10^8	1.077	0.100
Rhodococcus sp. PFS2.95	0	1×10^7	0.118	1×10^8	0.649	0.097	3.67×10^8	1.041	0.046
	0.5		0.116	7×10^7	0.719	0.072	3.67×10^8	1.069	0.071
	1		0.132	2×10^8	0.656	0.112	2.5×10^8	1.131	0.015
	2		0.142	2×10^8	0.677	0.106	3.67×10^8	1.096	0.037

	3		0.117	8 x 10^7	0.596	0.080	1.17 x 10^8	1.065	0.015
Rhodococcus sp. PFS3.70	0	4 x 10^6	0.131	5 x 10^7	0.558	0.107	2.17 x 10^8	0.976	0.063
	0.5		0.122	9 x 10^7	0.588	0.133	1 x 10^8	1.046	0.004
	1		0.132	1 x 10^8	0.506	0.145	7.33 x 10^8	0.951	0.076
	2		0.142	8 x 10^7	0.534	0.128	2 x 10^8	0.881	0.039
	3		0.091	9 x 10^7	0.507	0.135	1.33 x 10^8	0.775	0.015

Table S2. Growth of *Rhodococcus* sp. PFS1.20, *Rhodococcus* sp. PFS2.95, and *Rhodococcus* sp. PFS3.70 strains in the presence of bile salts.

Isolate	Bile salt concentration, %	Day 0 (OD_{600})	Day 1 (OD_{600})	Day 2 (OD_{600})
Rhodococcus sp. PFS1.20	0%	0.065	0.488	1.023
	0.25%	0.078	0.052	0.02
	0.50%	0.057	0.028	0.009
	0.75%	0.055	0.038	0.029
	1%	0.056	0.022	0.015
Rhodococcus sp. PFS2.95	0%	0.057	0.722	0.92
	0.25%	0.062	0.041	0.028
	0.50%	0.055	0.018	0.01
	0.75%	0.05	0.034	0.018
	1%	0.048	0.032	0.005
Rhodococcus sp. PFS3.70	0%	0.057	0.488	0.921
	0.25%	0.062	0.036	0.015
	0.50%	0.058	0.032	0.01
	0.75%	0.05	0.034	0.004
	1%	0.033	0.012	0.006
